# Pharmacological Inhibition of S100A4 Attenuates Fibroblast Activation and Renal Fibrosis

**DOI:** 10.3390/cells11172762

**Published:** 2022-09-05

**Authors:** Jia Wen, Baihai Jiao, Melanie Tran, Yanlin Wang

**Affiliations:** 1 Division of Nephrology, Department of Medicine, University of Connecticut School of Medicine, Farmington, CT 06030-1405, USA; 2 Division of Nephrology, Guangdong Provincial People’s Hospital, Guangdong Academy of Medical Sciences, Guangzhou 510080, China; 3 Department of Cell Biology, University of Connecticut School of Medicine, Farmington, CT 06030-1405, USA; 4 Renal Section, Veterans Affairs Connecticut Healthcare System, West Haven, CT 06516-2770, USA

**Keywords:** Smad3, S100A4, fibroblast, renal fibrosis

## Abstract

The TGF-β/Smad3 signaling pathway is an important process in the pathogenesis of kidney fibrosis. However, the molecular mechanisms are not completely elucidated. The current study examined the functional role of S100A4 in regulating TGF-β/Smad3 signaling in fibroblast activation and kidney fibrosis development. S100A4 was upregulated in the kidney in a murine model of renal fibrosis induced by folic acid nephropathy. Further, S100A4 was predominant in the tubulointerstitial cells of the kidney. Pharmacological inhibition of S100A4 with niclosamide significantly attenuated fibroblast activation, decreased collagen content, and reduced extracellular matrix protein expression in folic acid nephropathy. Overexpression of S100A4 in cultured renal fibroblasts significantly facilitated TGF-β1-induced activation of fibroblasts by increasing the expression of α-SMA, collagen-1 and fibronectin. In contrast, S100A4 knockdown prevented TGF-β1-induced activation of fibroblast and transcriptional activity of Smad3. Mechanistically, S100A4 interacts with Smad3 to stabilize the Smad3/Smad4 complex and promotes their translocation to the nucleus. In conclusion, S100A4 facilitates TGF-β signaling via interaction with Smad3 and promotes kidney fibrosis development. Manipulating S100A4 may provide a beneficial therapeutic strategy for chronic kidney disease.

## 1. Introduction

Chronic kidney disease (CKD) is a leading public health concern in all nations. Of the world’s population, more than 10% have CKD, with an occurrence rate of higher than 50% observed in high-risk populations [1,2]. The pathogenesis of renal fibrosis is demonstrated by histopathological activation of fibroblasts and extracellular matrix deposition, which in turn results in end stage kidney disease [3]. Therefore, a greater understanding the molecular mechanisms leading to kidney fibrosis development may provide novel therapeutic strategies for CKD.

Accumulating studies have established transforming growth factor-β1 (TGF-β1) as a major pathogenic factor that promotes renal fibrosis [4,5,6,7]. TGF-β1 binds to the TGF-β receptor II and activates TGF-β receptor I, which leads to Smad2 and Smad3 phosphorylation. Phosphorylated Smad2/3 then bind with Smad4, which enables this complex to translocate to the nucleus to activate its target genes [8,9,10]. Although the TGF-β1/Smad3 signaling pathway could be directly targeted to impede fibrosis development, TGF-β1/Samd3 has been reported to regulate other biological functions, including cell differentiation, immune response, autophagy and apoptosis [5,11]. Moreover, inhibition of TGF-β1/Smad3 could stimulate autoimmune diseases [5]. Consequently, blocking TGF-β1/Smad3 itself might not be feasible. Therefore, targeting cell-type specific Smad3 interacting proteins may provide a surrogate measure to prevent kidney fibrosis.

S100A4, also known as metastasin (Mst1) or fibroblast specific protein (FSP1), is a member of the S100 calcium binding protein family [12]. S100A4 has been extensively studied in cancer studies including colorectal [13], breast [14], lung [15] and liver cancers [16]. It is highly expressed in tumor cells and its expression is closely associated with cancer proliferation, invasion, and metastasis [17]. S100A4 has no known enzymatic activity; consequently, S100A4 induces its functional effects by associating with other proteins to regulate its target protein function. For example, S100A4 interacts with myosin-IIA to increase cellular motility [18]. Additionally, S100A4 induces degradation of p53 via its interaction with p53 to inhibit cisplatin-induced apoptosis [19]. More recently, S100A4 has been demonstrated to play a role in the pathogenesis of fibrotic disease [20]. Specifically, S100A4 expression was upregulated in a murine model of cardiac hypertrophy and heart failure through its interaction with p53 to promote cell proliferation and collagen deposition [21]. However, the role of S100A4 in renal fibrosis is not known, and whether S100A4 could affect Smad3 signaling remains unclear.

The current study demonstrates the role of S100A4 on the TGF-β1/Smad3 signaling pathway in fibroblast activation and demonstrates that S100A4 interacts with Smad3. Therefore, we examined the role of S100A4 in fibroblast activation and the development of renal fibrosis in vivo. In cultured kidney fibroblasts, we show that S100A4 functions as a coactivator of TGF-β1/Smad3 to promote fibroblast activation. Furthermore, S100A4 is induced in the kidney in folic acid nephropathy while pharmacological inhibition of S100A4 suppresses activation of fibroblast and attenuates the progression of renal fibrosis. Therefore, S100A4, acting as a Smad3 cofactor, facilitates the induction of the TGF-β1/Smad3 signaling pathway in fibroblasts and contributes to the progression of kidney fibrosis.

## 2. Materials and Methods

### 2.1. Reagents, Antibodies, and Plasmids

The antibodies used in the study were as follows: anti-GAPDH (1:5000, EMD Millipore, MAB374, Billerica, MA, USA), anti-Lamin A/C (1:400, Santa Cruz Biotechnology, sc-7293, Dallas, TX, USA), anti-Smad3 (1:1000, Cell Signaling Technology, 9523, Danvers, MA, USA), anti-phospho-Smad3 (Ser423/425) (1:1000, Cell Signaling Technology, 9520S), anti-Smad4 (1:400, Santa Cruz Biotechnology, sc-7966), anti-alpha-smooth muscle Actin (α-SMA) (1:500, Santa Cruz, sc-32251), anti-collagen type I (1:1000, SouthernBiotech, 1310-01, Birmingham, AK, USA), anti-fibronectin (1:1000, Sigma-Aldrich, F3648, St. Louis, MO, USA), anti-S100A4 (1:1000, Boster Bio, M01217-1, Pleasanton, CA, USA), anti-FLAG (1:100, GenScript, A00187, Nanjing, China), anti-GFP (1:100, GenScript, A01703), anti-mouse IgG (Cell Signaling Technology, 5415S) and anti-Rabbit IgG (Cell Signaling Technology, 2729S). These control antibodies were diluted to the same concentration as the specific antibody used in the analysis. The secondary antibodies used were donkey anti-mouse IgG (1:5000, MilliporeSigma, AP192P, Burlington, VT, USA), donkey anti-rabbit IgG (1:5000, MilliporeSigma, AP182P), donkey anti-goat IgG (1:5000, MilliporeSigma, AP180P), Alexa Fluor 488 donkey anti-mouse IgG (1:400, Invitrogen, A21202, Carlsbad, CA, USA) and Alexa Fluor 594 donkey anti-rabbit IgG (1:400, Invitrogen, A21201). Recombinant human TGF-β1 was obtained from PeproTech (100-21C), and the S100A4 inhibitor, Niclosamide (NLS, N3510) and Cremophor EL (238470) were obtained from Sigma (St. Louis, MO, USA).

The plasmid pGL3-SMA (−2555 + 2813 bp) was kindly provided by Dr. Jiliang Zhou (Medical College of Georgia, Augusta, GA, USA), and CMV-Renilla was kindly gifted by Dr. Liping Zhang (Baylor College of Medicine, Houston, TX, USA). pBV-SBE4-Luciferase (#16495) and pRK5-Smad3 (Flag-Smad3, #12625) plasmids were obtained from Addgene (Watertown, MA, USA).

### 2.2. Animal Experiments

C57BL/6 mice were obtained from the Jackson Laboratory (Bar Harbor, ME, USA). The animal experiments were conducted according to the guidelines from the National Institutes of Health Guide for the Care and Use of Laboratory Animals. All procedures were approved by the Institutional Animal Care and Use Committee of the University of Connecticut Health Center. Male mice (8 to 10 weeks old) weighing 20 to 25 g were used in the study. The mice were administered an intraperitoneal injection of folic acid (FA, 250 mg/kg dose) (Sigma) dissolved in NaHCO_3_ [22,23,24]. The control mice were administered an intraperitoneal injection of vehicle (0.3 mol/L NaHCO_3_). All the mice were euthanized at day 14 following FA or vehicle administration. To investigate the role of niclosamide in renal fibrosis, male C57BL/6J mice were randomly assigned to one of three groups: (i) Control (*n* = 5); (ii) FA (*n* = 5); (iii) FA + NLS (*n* = 5). FA was administered as described above. The FA + NLS group received a daily intraperitoneal injection of NLS (30 mg/kg per day) for 7 days starting at day 7 after FA administration. Niclosamide was made up in 10% Cremophor EL and 0.9% NaCl solution. All mice were euthanized at day 14 following FA injection.

### 2.3. Histological Examination and Immunohistochemistry

Paraffin embedded kidney sections were dewaxed and rehydrated with ethanol, and then stained with H&E to evaluate kidney morphology, and collagen content was determined by Sirius Red staining [25,26].

For immunohistochemistry staining, antigens were retrieved using citrate buffer (pH 6). Quenching of endogenous peroxidase activity was performed using 3% hydrogen peroxide for 10 min. The kidney sections were blocked with blocking solution for 1 h and then incubated with the primary antibody overnight at 4 °C. The following day, the kidney sections were incubated with the appropriate HRP-conjugated secondary antibody followed by incubation with ABC solution (Vector Laboratories, Newark, NJ, USA). The immunoreactivities were detected by diaminobenzidine (DAB) solution (Vector Laboratories). Finally, the sections were counterstained with hematoxylin. The immunostaining was examined by a microscope equipped with a digital camera [27,28]. The number of positive cells were qualified using Image Pro-Plus in a blinded manner.

### 2.4. Cell Culture

Normal rat kidney fibroblasts (NRK-49F, ATCC, Manassas, VA, USA) and mouse embryonic fibroblasts (NIH-3T3, ATCC) were grown in Dulbecco’s modified Eagle’s medium (DMEM, Thermo Fisher Scientific, Waltham, MA, USA) containing 5% fetal bovine serum (FBS) and 1% antibiotics [29,30]. Human embryonic kidney (HEK) 293T (ATCC) cells were cultured in DMEM with 10% FBS and 1% antibiotics. The cells were maintained in an incubator at 37 °C with 5% CO_2_. The NRK-49F and NIH-3T3 cells were incubated overnight in starvation media containing DMEM and 0.1% FBS before TGF-β1 (Peprotech, Rocky Hill, NJ, USA) treatment.

### 2.5. Overexpression and Knockdown Experiments

To overexpress S100A4, cells were seeded onto a 6-well plate in DMEM supplemented with 10% FBS, and then the cells were transfected with pEGFP or EGFP-S100A4 plasmids via electroporation following the manufacturer’s protocol (Neon Transfection System, Invitrogen). The transfection efficiency was determined by a fluorescence microscope.

To knockdown S100A4, S100A4 guide RNA (sgRNA) or control guide RNA (Con sgRNA) was synthesized. Lentiviral particles were created according to the manufacturers’ instructions (https://www.addgene.org/tools/protocols/plko/, accessed on 1 July 2019). The NRK-49F cells were seeded onto 12-well plates and then transfected with 500 µL of S100A4 sgRNA or Con sgRNA lentiviral particle solution using lipofectamine 2000 Reagent (Invitrogen). The following day, the cells were selected with puromycin (8 ug/mL) and knockdown of S100A4 was confirmed by Western blot analysis at 96 h post-transfection.

### 2.6. Luciferase Reporter Assay

NRK-49F cells were seeded onto 24-well plates in DMEM containing 5% FBS without antibiotics for 24 h before transfection. The transfection of cells with the SBE4-luc plasmid or pGL3-SMA-luc promoter plasmid was performed using electroporation as per the manufacturer’s instructions. All the samples were set up in triplicate and the transfection efficiency was normalized by co-transfection with pCMV-Renilla plasmid. The cells were subjected to overnight serum starvation with 0.1% FBS in DMEM. The cells were treated with TGF-β1 (2 ng/mL) or vehicle for 24 h and then harvested in 1× Passive Lysis Buffer. A Dual-Luciferase Reporter Assay System was used to determine luciferase activity (Promega, Madison, WI, USA).

### 2.7. Protein Immunoprecipitation

HEK293T cells were co-transfected with GFP-S100A4 and FLAG-Smad3 using Lipofectamine 2000 reagent. 293T cells at 48 h post-transfection and non-transfected NRK-49F and NIH-3T3 cells were collected and lysed in IP lysis buffer (25 mM Tris-HCl pH 7.4, 150 mM NaCl, 1% NP-40 and 5% glycerol) with the addition of phosphatase and protease inhibitors. The cell extracts were pre-cleared with protein A/G bead slurry for 1 h at 4 °C and then the supernatants were immunoprecipitated with anti-Smad3 antibody overnight at 4 °C. Rabbit normal IgG was used as the control. Protein A/G beads were added to the cell extracts and the extract-bead mixtures were incubated under rotary agitation for 2 h at 4 °C. The beads were washed extensively in IP buffer and the eluted samples were run on an SDS-PAGE gel.

### 2.8. Nuclear and Cytoplasmic Protein Extraction

Cells were lysed and then resuspended in hypotonic buffer (10 mM HEPES pH 8.0, 1.5 mM MgCl_2_, 10 mM KCL). The cell extracts were centrifuged at 1250 rpm for 5 min at 4 °C to pellet the cells. Cytoplasmic lysis buffer (hypotonic buffer plus 0.1 mM EDTA and 0.5% NP-40) was used to resuspend the pellet. The extracts were centrifuged and the supernatant was kept as the cytoplasmic fraction while the pellet was washed with hypotonic buffer. The pellet was resuspended in nuclear lysis buffer (10 mM HEPES pH 8.0, 1.5 mM MgCl_2_, 400 mM NaCl, 0.1 mM EDTA, 20% glycerol), followed by incubation for 30 min on ice. The suspension was sonicated on ice for 15 s to release the nuclear proteins. The cell lysates were then centrifuged at 12,000 rpm for 15 min at 4 °C, and the supernatant collected was retained as the nuclear fraction.

### 2.9. Western Blot Analysis

Total proteins were isolated from cells and kidney tissue using a radioimmunoprecipitation assay buffer (RIPA) buffer as described previously [26,31]. The proteins were quantified using a BCA protein assay kit and then separated on an SDS-PAGE gel. After the transfer of proteins onto a nitrocellulose membrane, the membrane was blocked in 5% milk. The membranes were incubated with primary antibodies overnight followed by incubation with the corresponding HRP-conjugated secondary antibodies. The proteins of interest were captured using a ChemiDoc MP Imaging System (Bio-Rad Laboratories, Hercules, CA, USA) and the signal intensities were determined using NIH ImageJ software.

### 2.10. Immunofluorescence Staining

Cells were cultured on glass coverslips, and then fixed using 4% formaldehyde. The cells were permeabilized with 0.25% Triton-X 100. Non-specific binding was blocked with BlockAid blocking solution (B10710, Invitrogen) and then the cells were incubated with the indicated antibody overnight at 4 °C. The corresponding fluorescent secondary antibody was used following manufacturer’s instructions (Invitrogen). The coverslips containing the cells were inverted on glass slides in mounting medium containing DAPI (H-1500, Vector Laboratories, Burlingame, CA, USA). The cells were examined using a confocal microscope (Zeiss LSM 880, Oberkochen, Germany).

### 2.11. Statistical Analysis

The results are expressed as mean ± SEM. A two-tailed unpaired Student’s t-test was used to determine differences between two groups. A one-way ANOVA with Bonferroni correction was used to determine differences across multiple groups. SPSS 22.0 (SPSS Inc., Chicago, IL, USA) was used to perform the statistical analyses, and statistical significance was established as having a *p* value less than 0.05.

## 3. Results

### 3.1. S100A4 Is Upregulated in the Kidney of Folic Acid Nephropathy

**Figure 1:**
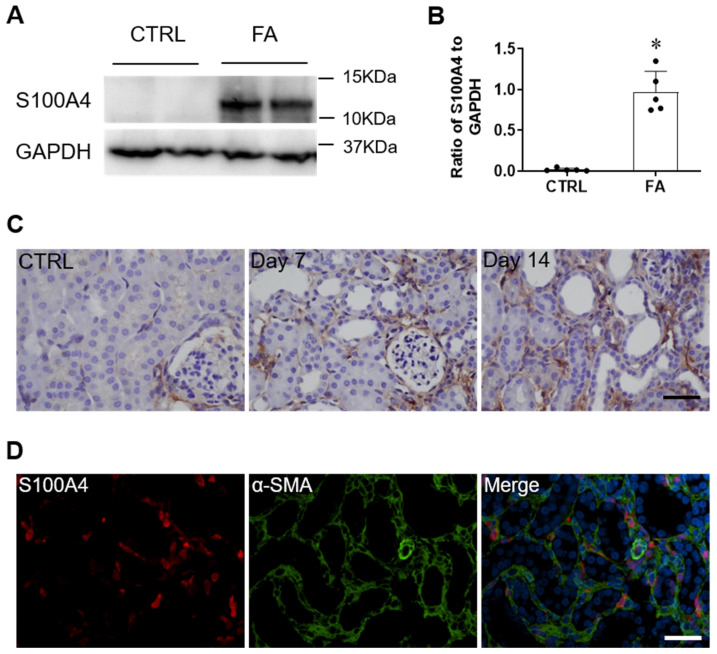
S100A4 is upregulated in the kidney with folic acid nephropathy. (**A**) Representative Western blots show protein levels of S100A4 in the kidneys with FA treatment. (**B**) Quantitative analysis of S100A4 protein levels in the kidneys with FA treatment. Data are expressed as Mean ± SEM, *n* = 5 per group. * *p* < 0.05 vs. CTRL group. (**C**) Representative images of kidney sections immunostained for S100A4 (brown) and counterstained with hematoxylin (blue). (**D**) Immunofluorescence staining showed the expression pattern of S100A4 and α-SMA at 7 days after folic acid treatment. Scale bar = 50 μm.

To determine whether S100A4 is upregulated in the kidney in vivo, we examined the protein levels of S100A4 in mice with FA nephropathy. Immunoblotting analysis demonstrated that S100A4 was significantly upregulated at day 14 following FA administration (Figure 1A,B). To evaluate the specific cell type that is responsible for S100A4 induction in kidneys with FA injury, sections were immunostained for S100A4. The results revealed that S100A4-positive staining was localised in the interstitial cells of the kidney at day 7 and was further elevated at day 14 following FA administration (Figure 1C). To examine if S100A4 is induced in activated fibroblasts, kidney sections were stained for S100A4 and α-SMA, a marker of activated fibroblasts. The results of double-immunofluorescence staining showed that S100A4-positive staining was colocalized with α-SMA, indicating that S100A4 is mainly induced in myofibroblasts (Figure 1D).

### 3.2. Pharmacologic Inhibition of S100A4 Reduces Renal Fibrosis in Folic Acid Nephropathy

**Figure 2:**
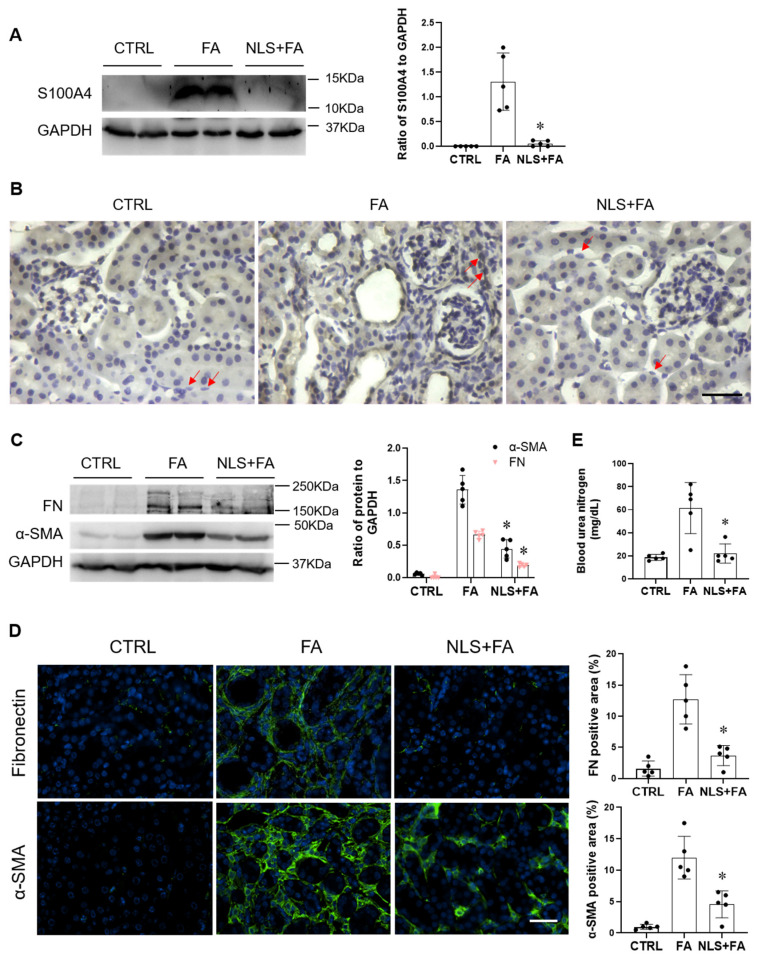
Niclosamide ameliorates renal fibrosis in folic acid nephropathy. (**A**) Western blots show protein levels of S100A4 in the kidneys of mice at day 14 after FA administration (left panel), and quantitative analysis of S100A4 protein levels in the kidneys of mice with FA administration (right panel). (**B**) Representative images of kidney sections immunostained for Smad3 (brown) and counterstained with hematoxylin (blue) in mice administered with vehicle (CTRL), FA, or Niclosamide (NLS) + FA. Red arrow indicates the Smad3 staining in the nucleus of tubulointerstitial cells. (**C**) Western blots show α-SMA and fibronectin protein levels in the kidneys at day 14 after CTRL, FA and NLS + FA administration (**left**). Quantitative analysis of fibronectin and α-SMA-positive cells in the kidneys of mice administered with CTRL, FA, or NLS + FA (**right**). (**D**) Representative images of fibronectin and α-SMA expression in kidney of mice administered with CTRL, FA, or NLS + FA (left panel). Quantitative analysis of fibronectin and α-SMA positive cells in kidney sections of mice administered with CTRL, FA, or NLS + FA (right panel). Scale bar = 50 μm. (**E**) Blood urea nitrogen (BUN) levels in mice at day 14 after CTRL, FA and NLS + FA administration. Data are expressed as Mean ± SEM, *n* = 5 per group. * *p* < 0.05 vs. FA group.

To further establish the role of S100A4 in kidney fibrosis in vivo, we investigated the effects of the S100A4 inhibitor niclosamide on S100A4 expression in mice with renal fibrosis. C57BL/6 mice were injected intraperitoneally with folic acid and after 7 days, mice were injected daily with niclosamide for 7 days. The protein level of S100A4 was elevated in the FA group compared with that of control group (Figure 2A), whereas niclosamide administration significantly attenuated S100A4 expression level in the kidney with FA nephropathy (Figure 2A).

We next evaluated whether niclosamide could affect Smad3 signaling in vivo. Kidney sections of mice with FA nephropathy were immunostained for Smad3. The results revealed a marked upregulation of Smad3 in the nucleus of tubulointerstitial cells, whereas administration of niclosamide significantly diminished Smad3 induction (Figure 2B). To determine the biological function of S100A4 inhibition in mice with kidney fibrosis, an immunoblotting analysis was carried out to assess α-SMA and fibronectin levels. Mice injected with folic acid had considerably more fibronectin and α-SMA compared with vehicle treated controls (Figure 2C). In contrast, inhibition of S100A4 with niclosamide markedly attenuated fibronectin and α-SMA levels with FA nephropathy (Figure 2C). These data were further established by fluorescence staining in the kidney; a higher percentage of α-SMA-positive and fibronectin-positive areas were observed in FA nephropathy which were reduced with niclosamide administration (Figure 2D). These results indicate that inhibition of S100A4 with niclosamide alleviates kidney fibrosis in part by preventing Smad3 signaling.

To determine the effect of niclosamide administration on renal function, levels of blood urea nitrogen (BUN) were measured. Levels of BUN were higher in the FA treated group compared with the control group, which was markedly reduced by niclosamide (Figure 2E). Our results demonstrate that niclosamide preserves renal function in mice treated with folic acid.

### 3.3. S100A4 Interacts with Smad3 under Physiological Conditions

**Figure 3:**
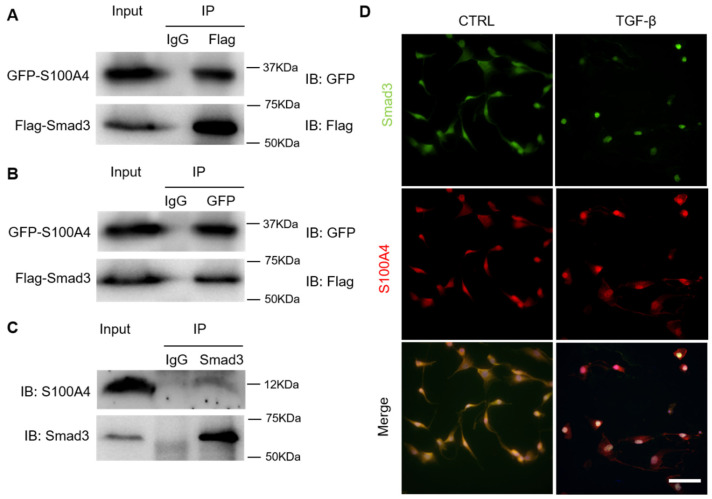
S100A4 interacts with Smad3 under physiological conditions. HEK-293T cells were transfected with GFP-S100A4 or FLAG-Smad3 plasmids. (**A**) Cell lysates were immunoprecipitated with anti-Flag antibody and analyzed by Western blot with the indicated antibodies. (**B**) Cell lysates were immunoprecipitated with anti-GFP antibody and analyzed by Western blots with the indicated antibodies. (**C**) Western blots show endogenous interaction of S100A4 and Smad3 in NRK-49F cells. Cell lysates were immunoprecipitated with an IgG antibody or a Smad3 antibody and analyzed by Western blot with the indicated antibodies. (**D**) Immunofluorescence analysis of Smad3 and S100A4 co-localization in NRK-49F cells. Cells were treated with TGF-β1 (2 ng/mL) for 1 h and then stained with an anti-Smad3 antibody (green), anti-S100A4 antibody (red), and counterstained with DAPI (blue). Scale bar = 50 μm.

Smad3 is a major transcriptional regulator of fibroblast activation. We identified S100A4 as a novel Smad3 interacting protein by proteomics analysis. To confirm the interaction of Smad3 with S100A4, we performed a co-immunoprecipitation assay. After transfection of HEK293T cells with GFP-tagged S100A4 and FLAG-tagged Smad3 plasmids, the cell extracts were immunoprecipitated with an antibody for anti-Flag and analyzed with anti-GFP antibody for S100A4 by Western blot analysis. The results show that GFP co-immunoprecipitated with anti-Flag (Figure 3A), indicating that Smad3 co-immunoprecipitated with S100A4. In a reciprocal experiment, S100A4 could interact with Smad3 (Figure 3B). To further confirm the specificity of the Smad3-S100A4 interaction, endogenous Smad3 protein in NRK-49F cells was immunoprecipitated with anti-Smad3. S100A4 was observed in the immunoprecipitates of anti-Smad3, indicating that endogenous S100A4 interacts with Smad3 in fibroblasts (Figure 3C). Additionally, we examined the co-localization of S100A4 and Smad3 in NRK-49F cells. Fluorescence images showed S100A4 was expressed in the nucleus and cytoplasm under rest conditions. The treatment of TGF-β1 promoted S100A4 translocation into the nucleus with Smad3 (Figure 3D).

### 3.4. S100A4 Promotes Smad3 Nuclear Translocation by Maintaining the Smad3/Smad4 Complex

**Figure 4:**
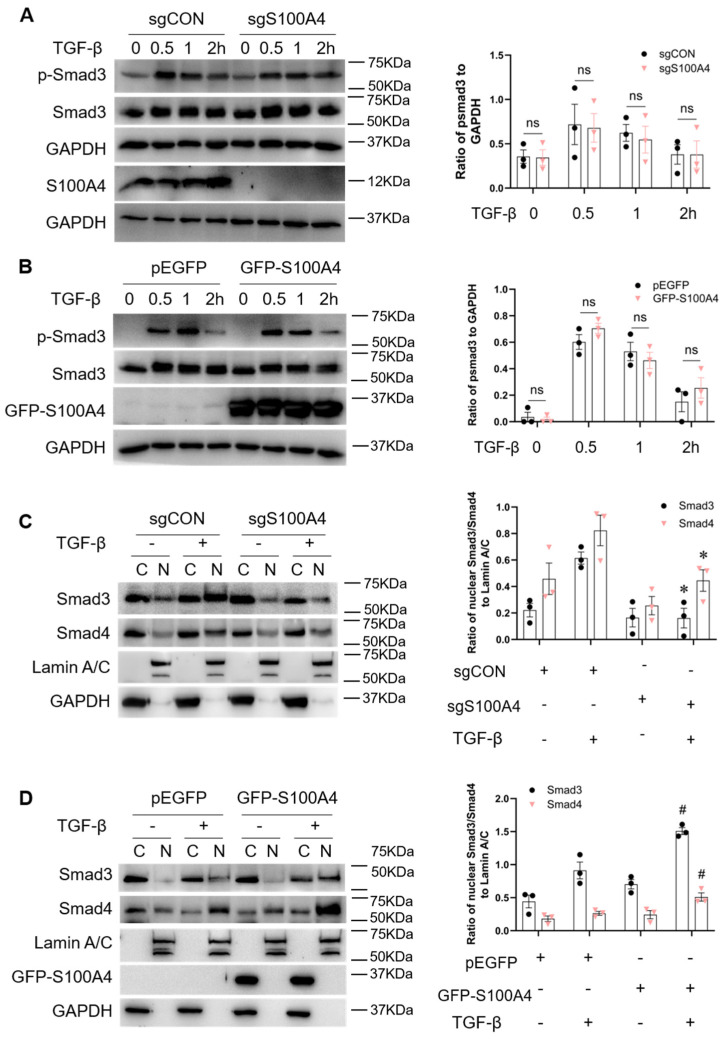
S100A4 promotes Smad3 nuclear translocation with TGF-β1 treatment. Western blots show phosphorylation of Smad3 in NRK-49F cells with (**A**) S100A4 knockdown and (**B**) Overexpression of S1004A treated with TGF-β1 (2 ng/mL) at various time points. (**C**) NRK-49F stable cells expressing S100A4 (sgS100A4) were treated with TGF-β1 (2 ng/mL) for 1 h, and cytoplasmic and nuclear fractions were prepared. Protein levels of Smad3 and Smad4 were determined by immunoblotting analysis. (**D**) NRK-49F cells transduced with pEGFP or GFP-S100A4 were treated with TGF-β1 (2 ng/mL) for 1 h, and cytoplasmic and nuclear fractions were prepared. Cell extracts were analyzed for protein levels of Smad3 and Smad4 by Western blot analysis. Data are expressed as Mean ± SEM of three independent experiments. * *p* < 0.05 vs. sgCON + TGF-β1 and ^#^ *p* < 0.05 vs. pEGFP + TGF-β1.

To understand the mechanism of how S100A4 regulates TGF-β1-induced fibroblast activation, we determined whether S100A4 affects Smad3 signaling, including phosphorylation, nuclear translocation, and Smad3/Smad4 complex formation. Knockdown of S100A4 with lentiviral vectors expressing sgRNA (sgS100A4) (Figure 4A) or overexpression of S100A4 with GFP-S100A4 (Figure 4B) did not affect TGF-β1 induced phosphorylation of Smad3. The data demonstrate that S100A4 occurs downstream of Smad3 phosphorylation.

Since S100A4 did not affect Smad3 phosphorylation, we speculate that S100A4 might regulate the cellular response of TGF-β1 by regulating Smad3 subcellular distribution. To this end, we separated nuclear and cytoplasmic fractions of NRK-49F cell lysates to investigate the role of S100A4 in Smad3 nuclear translocation. Upon TGF-β1 stimulation, knock down of S100A4 diminished the nuclear accumulation of Smad3 and reduced expression of Smad4 (Figure 4C), whereas overexpression of S100A4 increased Smad3 and Smad4 nuclear translocation (Figure 4D). Our data reveal that S100A4 positively regulates Smad3 and Smad4 nuclear translocation.

**Figure 5:**
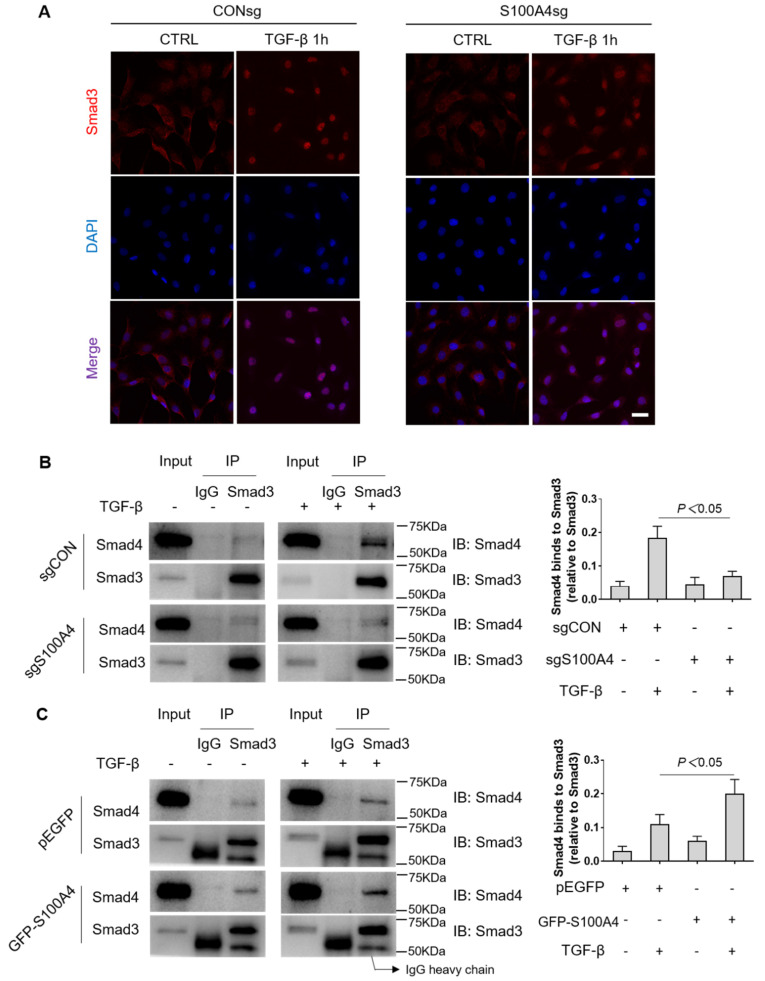
S100A4 promotes Smad3 nuclear translocation by stabilizing Smad3/Smad4 complex. (**A**) Representative images of immunofluorescence staining of Smad3 nuclear translocation in NRK-49F sgCON and sgS100A4 cells. Cells were treated with TGF-β1 (2 ng/mL) for 1 h and then stained with an anti-Smad3 antibody (red), and counterstained with DAPI (blue). (**B**) NRK-49F stable cells expressing CON sgRNA or S100A4 sgRNA were treated with TGF-β1 (2 ng/mL) for 1 h. Cell extracts were immunoprecipitated with anti-Smad3 antibody and analyzed by Western blot with an anti-Smad4 antibody. (**C**) NRK-49F cells transduced with pEGFP or GFP-S100A4 were treated with TGF-β1 (2 ng/mL) for 1 h. Extracts were immunoprecipitated with an anti-Smad3 antibody and analyzed by Western blot with an anti-Smad4 antibody. Data are expressed as Mean ± SEM of three independent experiments. Scale bar = 50 μm.

To further confirm the cellular localization of Smad3, immunofluorescence staining was performed in NRK-49F stable cells with S100A4 knockdown. Consistent with our previous observation, Smad3 was localized in the cytoplasm under basal conditions while TGF-β1 promoted Smad3 nuclear translocation (Figure 5A). Additionally, knockdown of S100A4 significantly blocked TGF-β1 induced Smad3 translocation into the nucleus (Figure 5A). Based on the above findings, we speculate that S100A4 could enhance nuclear translocation of Smad3 through stabilization of the Smad3/Smad4 complex. To confirm this, we examined the interaction of S100A4 with the Smad3/Smad4 complex. NRK-49F stable cells with S100A4 knockdown were treated with TGFβ1 for 1 h and immunoprecipitated with IgG or Smad3 antibody. As shown in Figure 3B, Smad3 associates with Smad4 following TGF-β1 treatment, whereas knockdown of S100A4 significantly inhibited the association of the Smad3/Smad4 complex with TGF-β1 treatment (Figure 5B). In contrast, overexpression S100A4 in NRK-49F cells increased TGF-β1 induced Smad3/Smad4 complex formation (Figure 5C). These results suggest that S100A4 promotes Smad3 nuclear translocation by maintaining the Smad3/Smad4 complex.

### 3.5. Knockdown of S100A4 Abrogates TGF-β1 Induced Fibroblast Activation

**Figure 6:**
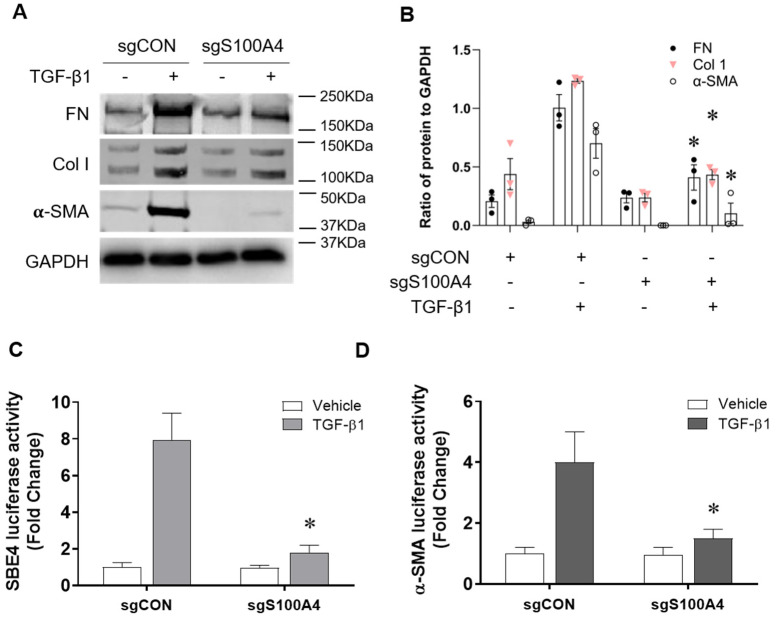
Knockdown of S100A4 inhibits fibroblast activation and TGF-β1 induced transcriptional activity. (**A**) Representative Western blots show knockdown of S100A4 reduced protein levels of fibronectin, collagen 1 and α-SMA in NRK-49F cells treated with TGF-β1 (2 ng/mL, 24 h). (**B**) Quantitative analysis of levels of collagen 1, α-SMA and fibronectin in NRK-49F cells. (**C**) Luciferase assay shows knockdown of S100A4 attenuates TGFβ-induced Smad3 promoter activity in NRK-49F cells. (**D**) Luciferase assay shows knockdown of S100A4 attenuates TGF-β1-induced α-SMA promoter activity in NRK-49F cells. Data are expressed as Mean ± SEM of three independent experiments. * *p* < 0.05 vs. sgCON + TGF-β1.

To establish the role of S100A4 in TGF-β1 induced activation of fibroblasts, we knocked down S100A4 with lentiviral vectors expressing sgRNA (sgS100A4) in NRK-49F cells. TGFβ induced ECM protein expression levels, including collagen-1 and fibronectin, and the myofibroblast marker α-SMA in NRK-49F cells. Moreover, knockdown of S100A4 attenuated ECM and α-SMA protein expression levels in TGF-β1 stimulated cells (Figure 6A,B). To assess the molecular mechanism by which S100A4 induces fibroblast activation, we determined whether S100A4 could affect Smad3 promoter activity. NRK-49F cells transduced with sgS100A4 or sgCON were transfected with Smad3 binding element-luciferase (SBE4-luc) plasmid, a synthetic TGF-β-responsive reporter gene. Smad3 luciferase reporter activity was increased in response to TGF-β1, while knockdown of S100A4 significantly decreased SBE4 luciferase reporter activity (Figure 6C). Fibroblast activation, leading to myofibroblasts is characterized by expression of α-SMA. Therefore, we evaluated whether knockdown of S100A4 affects Smad3-dependent α-SMA promoter activity. Similar to the SBE4-luciferase reporter assay, knockdown of S100A4 significantly suppressed α-SMA promoter activity in response to treatment with TGF-β1 (Figure 6D). Altogether, these results indicate that S100A4 promotes TGFβ-induced fibroblast activation via positive regulation of Smad3 transcriptional activity.

### 3.6. Overexpression of S100A4 Promotes TGF-β1-Induced Fibroblast Activation

**Figure 7:**
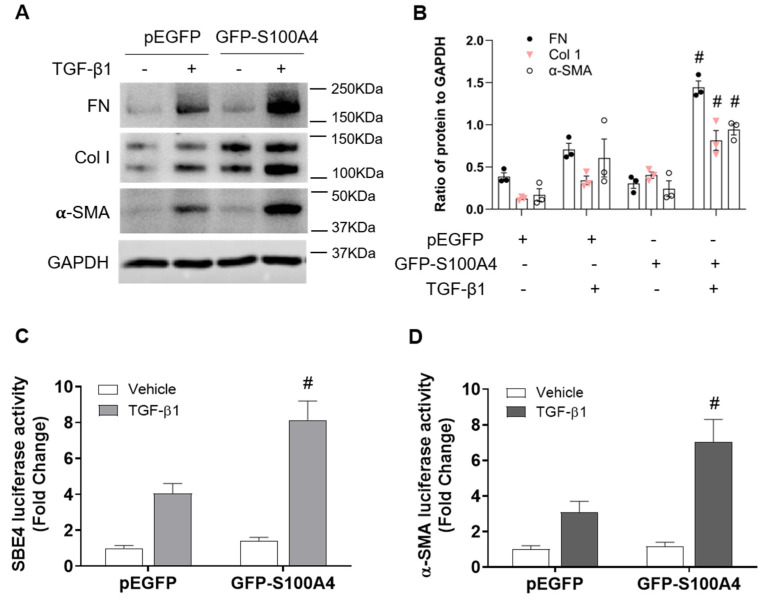
Overexpression of S100A4 stimulates fibroblast activation and TGF-β1-induced transcriptional activity. (**A**) Overexpression of S100A4 (GFP-S100A4) in NRK-49F cells shows enhanced protein levels of fibronectin, collagen 1 and α-SMA treated with TGF-β1 (2 ng/mL, 24 h). (**B**) Quantitative analysis of fibronectin, collagen 1 and α-SMA protein levels in NRK-49F cells. (**C**) Luciferase assay shows overexpression of S100A4 (GFP-S100A4) enhanced Smad3 promoter activity in NRK-49F cells. (**D**) Luciferase assay shows overexpression of S100A4 (GFP-S100A4) enhanced α-SMA promoter activity in NRK-49F cells. Data are expressed as Mean ± SEM of three independent experiments. ^#^ *p* < 0.05 vs. pEGFP + TGF-β1.

To firmly establish the role of S100A4 in fibroblast activation, we overexpressed S100A4 in NRK-49F cells using lentiviral vectors expressing GFP-S100A4. Overexpression of S100A4 promotes TGFβ1-induced fibroblast activation as indicated by increased expression of ECM proteins and α-SMA (Figure 7A,B). Additionally, in response to TGF-β1, overexpression of S100A4 enhanced Smad3 (Figure 7C) and α-SMA (Figure 7D) promoter activity. Our results demonstrate that S100A4 facilitates fibroblast activation via upregulation of Smad3 activity.

## 4. Discussion

Mounting evidence indicates that S100A4 has a pivotal role in fibrogenesis. Xia et al. [27,32] reported that in idiopathic pulmonary fibrosis, S100A4 promoted fibrogenic mesenchymal progenitor cells self-renewal through interacting with L-isoaspartyl methyltransferase. Nonetheless, the role of S100A4 in kidney fibrosis is not well established. Nishitani et al. [33] reported that the number of S100A4 positive fibroblasts were a significant determining factor for the development of end stage renal disease in patients with IgAN. In the current study, our findings have demonstrated that S100A4 modulates kidney fibrosis by regulating the TGF-β/Smad3 signaling pathway. This is supported by the following evidence. First, S100A4 interacts with Smad3 in renal fibroblasts in vivo and in vitro. Second, S100A4 facilitates the interaction of Smad3 and Smad4. Exogenous expression of S100A4 further promotes the formation of the S100A4/Smad3 complex while knockdown of S100A4 inhibits this formation, suggesting that S100A4 functions as a bridge for the engagement of Smad3 and Smad4. S100A4 then promotes Smad3/Smad4 translocation to the nucleus. Third, S100A4 is upregulated in renal fibroblasts during fibrogenesis development in FA nephropathy in mice. Finally, pharmacologic inhibition of S100A4 attenuates ECM proteins and expression of α-SMA in fibroblasts treated with TGFβ in vitro and in mice with FA nephropathy in vivo. In line with our study, Tomcik et al. [34] reported that TGF-β1 induced S100A4 expression, and enhanced fibroblast activation in systemic sclerosis most likely via the induction of the canonical TGF-β/Smad pathway, indicating that S100A4 and TGF-β/Smad can regulate each other to form a vicious cycle.

A greater understanding of the contribution of TGF-β1 signaling in kidney fibrosis has revealed other molecular targets, namely restoring the equilibrium between profibrotic Smad3 and antifibrotic Smad7 [5]. Our present study discovered S100A4 functioning as a Smad3 co-activator. Therefore, targeting S100A4 expression may provide an effective strategy for fibrotic diseases. In a xenograft SCID mouse model of human psoriasis, inhibition of S100A4 via a specific blocking antibody led to a significant reduction in epidermal thickness and impairment in cell proliferation [35]. Anti-S100A4 antibody also reduced the expression of type I collagen in both dermal fibroblasts and hepatic stellate cells [34,36]. Additionally, niclosamide, an approved antihelminthic drug, has been identified as a potential S100A4 transcription inhibitor [37]. A recent study demonstrated that phosphate niclosamide markedly attenuated interstitial fibrosis in a mouse model of adriamycin nephropathy [38]. Our study showed that niclosamide attenuated TGF-β1-induced fibroblast activation. Moreover, niclosamide treatment reduced S100A4 expression and fibrogenesis in FA nephropathy. It is noteworthy to mention that niclosamide is not specific for S100A4. Niclosamide has been shown to inhibit several profibrotic signaling pathways, including STAT3, AKT, and Wnt/β-catenin [39,40]. In this context, further studies are warranted to develop an agent that specifically targets S100A4 as an alternative anti-fibrotic strategy.

Several studies have reported that S100A4 interacts with a plethora of specific proteins to control their function. S100A4 has been shown to negatively regulate p53 downstream genes to promote cardiac fibroblast proliferation and collagen expression [21]. Li et al., showed that S100A4 interacts directly with myosin-IIA to enhance cellular directional motility [18]. It has also been reported that S100A4 can physically and functionally interact with Smad3 to increase TGF-β-induced matrix metalloproteinase-9 expression and invasion ability in breast cancer cells [41]. However, how S100A4 regulates TGF-β/Smad3 signaling is not known. At present, we have uncovered an unrecognized role of S100A4 in renal fibrosis. Our results demonstrate that S100A4 specifically binds to Smad3. This interaction enables Smad3 to combine with Smad4, thereby stabilizing the Smad3/Smad4 complex to promote TGF-β1 mediated Smad3-dependent fibrotic protein expression and transcriptional activity. Furthermore, our results demonstrate that pharmacological inhibition of S100A4 attenuates the development of fibrogenesis in a murine model of FA nephropathy. We provide the first evidence that S100A4 has a crucial role in regulating the TGF-β/Smad3 signaling pathway and may be associated with the development of kidney fibrosis.

Previous studies have reported a close association between S100A4 and the TGF-β signaling pathway in tumorigenesis and Crohn’s disease. Li et al. [42] showed that S100A4 facilitates TGF-β induced epithelial mesenchymal transition by enhancing Smad2 phosphorylation in gastric cancer cells. Intriguingly, TGF-β1 treatment also stimulated S100A4 expression, which suggests a TGF-β/Smad2-S100A4 positive feedback loop. Moreover, Wang et al. [43] showed that S100A4 was required in a LASP1 mediated TGFβ1 induced EMT process in human colorectal cancer, while depletion of S100A4 suppressed the phosphorylation status of MEK, ERK, AKT, and Smad2, which indicates that S100A4 plays a key role in TGF-β1 induced cell invasiveness. Additionally, Cunningham et al. [44] showed that S100A4 can promote intestinal fibroblasts migration from patients with Crohn’s disease, possibly by promoting Smad3 activation. However, the underlying mechanism that S100A4 potentiates the TGF-β1 signaling pathway remains elusive. Matsuura et al. [41] showed that S100A4 directly interacts with the N-terminal domain of Smad3, which potentiated the transcription activity of Smad2/3 in cancer cells. Our data demonstrate that S100A4 promotes TGF-β1 induced activation of renal fibroblasts and transcription activity of Smad3; therefore, we investigated whether these effects were due to S100A4-Smad3 interaction. In cultured HEK-293T cells co-transfected with GFP-S100A4 and Flag-Smad3, we discovered that S100A4 interacts with Smad3. We provide further evidence that S100A4 physiologically associate with Smad3 in renal fibroblasts.

S100A4 was first identified as a fibroblast specific protein 1 (FSP1) [45]. A subsequent study showed that FSP1-positive fibroblasts are derived from bone marrow and tubular epithelial cells [46]. While Smad3 is a central mediator of the TGF-β1 signaling pathway in fibrosis, the role of Smad2 in the kidney is less well understood. Meng et al. have shown that conditional Smad2 disruption in tubular epithelial cells of the kidney promotes fibrosis through upregulation of TGF-β1/Smad3 signaling in mice with obstructive injury [47]. Additionally, Loeffler et al. have demonstrated that S100A4 (FSP1)-specific Smad2 knockout in the kidney attenuates fibrosis and mitigates epithelial-to-mesenchymal transition in mice with diabetic nephropathy induced by STZ through suppression of TGF-β1 and Smad3 protein expression [48]. It would be interesting to investigate whether S100A4 plays a role in regulating Smad2 activity.

In summary, we identified S100A4 as a novel profibrotic factor in the development of renal fibrosis. The current studies demonstrate that S100A4 promotes TGF-β1-induced fibroblast activation by interacting with Smad3 and increasing Smad3/Smad4 complex formation, which facilitates their nuclear translocation. Therefore, S100A4 may function as an alternative target for the prevention of chronic kidney disease.

## Data Availability

The data that support the findings of this study are available from the corresponding author upon reasonable request.

## Abbreviations

BUN	serum urea nitrogen
CKD	chronic kidney disease
COL-1	collagen type I
ECM	extracellular matrix
EMT	epithelial mesenchymal transition
FBS	fetal bovine serum
FA	folic acid
FN	Fibronectin
NRK-49F	Normal rat kidney fibroblast
TGF-β1	transforming growth factor β1
α-SMA	alpha-smooth muscle actin.
